# Supplementary material for: hDNA2 nuclease/helicase promotes centromeric DNA replication and genome stability
Source: EMBO J. 2018 May 17;37(14):e96729. doi: 10.15252/embj.201796729 (PMC6043852; doi:10.15252/embj.201796729)
Supplement: Supplementary file 1 — Expanded View Figures PDF [file EMBJ-37-e96729-s001.pdf]

Expanded View Figures

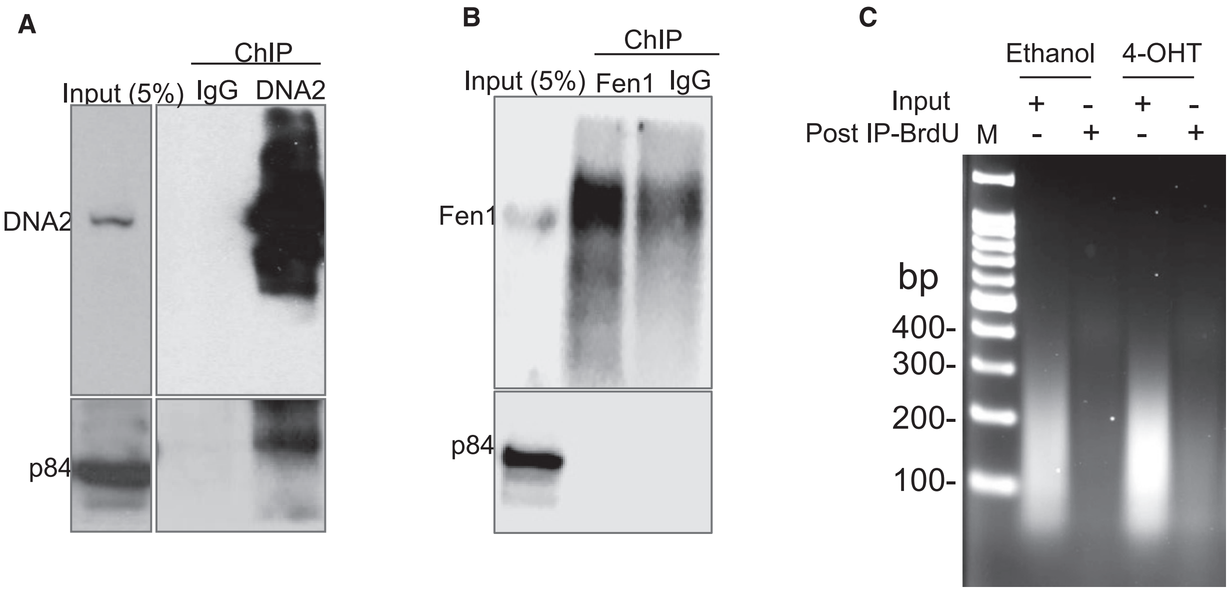

Figure EV1. Controls for generating DNA libraries analyzed by whole-genome sequencing.

A, B Western blot analysis to show efficiency of the DNA2 (A) and (B) Fen1 antibodies used in ChIP. Blots were probed for p84 to confirm specificity.

C Isolation of BrdU-negative DNA for whole-genome sequencing. $DNA2^{Flox/-/-}$ cells were treated with 4-OHT for 24 h, followed by incubation with 10 μM BrdU for 32 h. Cells were then fixed and sonicated, DNA was extracted, and BrdU-labeled DNA fragments were depleted. DNA samples from the indicated cells before (input) and after depletion of BrdU-labeled DNA were electrophoresed using a 2% agarose gel and stained with ethidium bromide.

Source data are available online for this figure.

© 2018 The Authors

Motifs	Peaks (%)[1]	Mean sites /peak	P value[2]
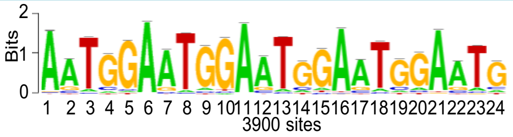	70 (26.5)	66.77	1.2e-88
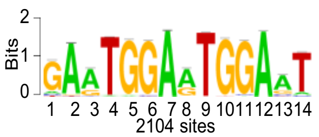	72 (27.3)	45.64	3e-85
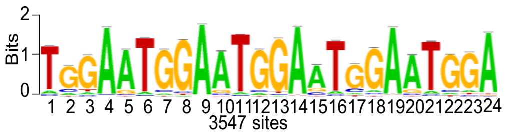	70 (26.5)	66.30	3.3e-42
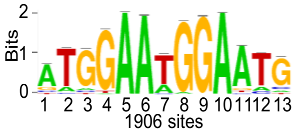	75 (28.4)	37.05	1.9e-34
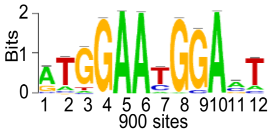	75 (28.4)	32.56	5.9e-33
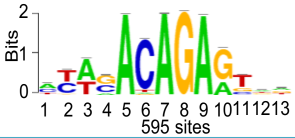	182 (68.9)	2.95	9.5e-23
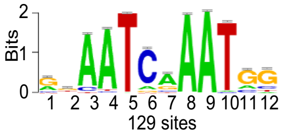	71 (26.9)	23.30	1.4e-20
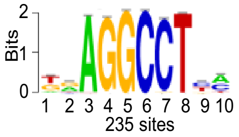	88 (33.3)	2.08	2.7e-19
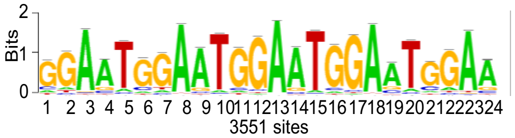	70 (26.5)	66.46	4.4e-18
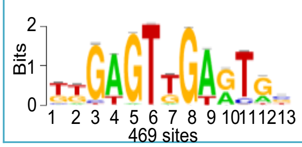	201 (76.1)	3.89	2.7e-16

Figure EV2. The top 10 motifs identified from ChIP-seq.

[1]Percentage of peaks containing the motif and their average occurrence in the peaks. [2]The presence of motifs was ranked by their *P*-values that were generated by Fisher's exact test.

© 2018 The Authors

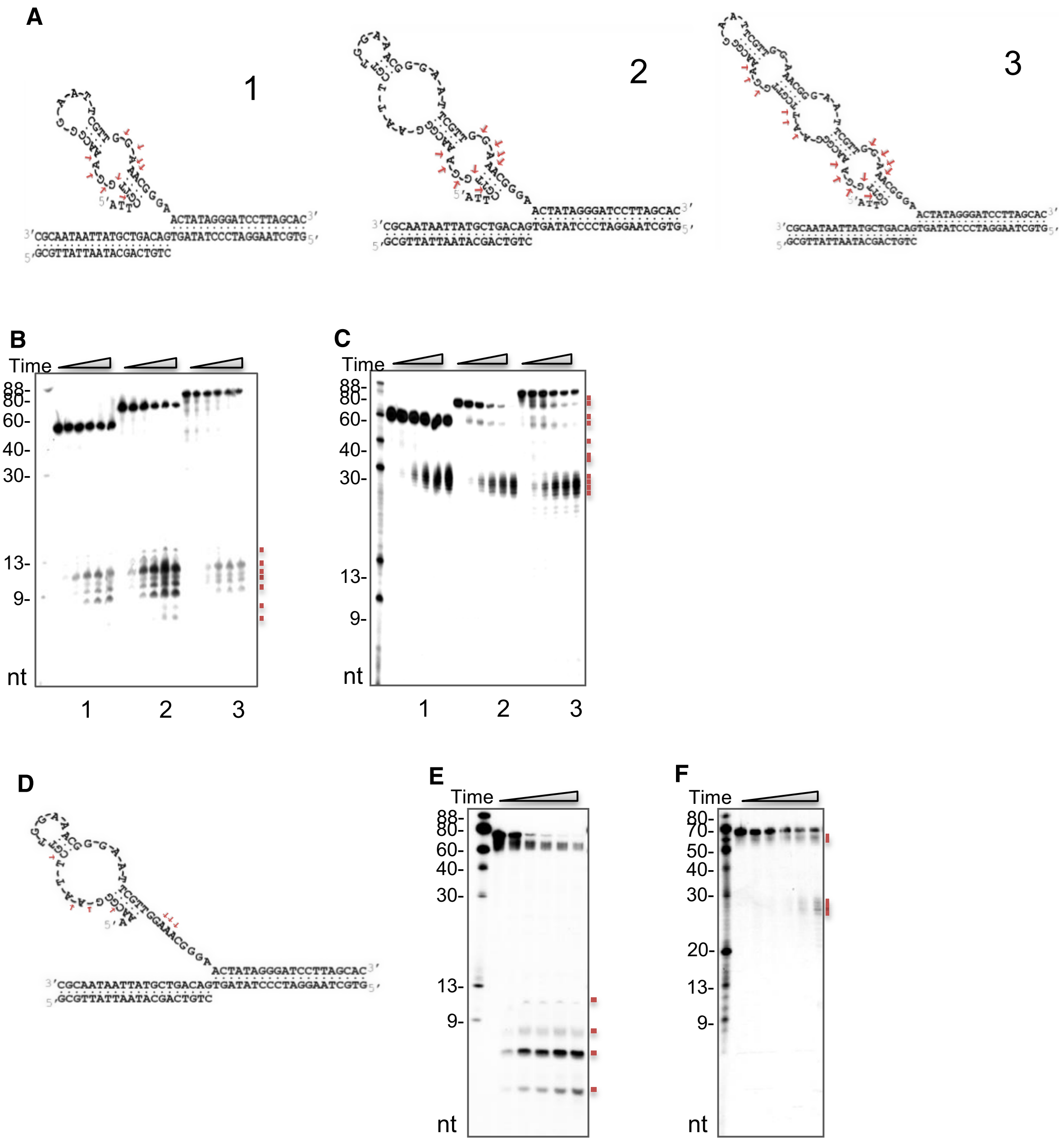

Figure EV3. DNA2 cleaves at the 5′ end of each complementary DNA where the loops are.

A Schematic graphs show the predicted structures of each substrate from the RNAfold software package. The cleavage signatures of DNA2 on these substrates are shown by arrows.

B, C DNA2 activity on substrates with similar structures as those shown in (A). Three substrates were made from two (substrate 1), three (substrate 2), or four repeats (substrate 3) of the 17-nt CENP-B-boxes to mimic the α-satellite stem-loop structure. WT DNA2 (5 ng) was incubated with 1 pmole of (B) 5′- or (C) 3′-radiolabeled substrate for 5, 10, 20, 30, or 40 min. Each small red square marks a cleavage product.

D–F An intermediate DNA product (illustrated in D) was specifically designed to determine if the second loop of substrate 2, shown in panel (A), was preferentially cleaved by DNA2. WT DNA2 (5 ng) was incubated with the (E) 5′- or (F) 3′-radiolabeled substrates for the same amount of time as in panels (B and C) to assess the cleavage.

Source data are available online for this figure.

© 2018 The Authors

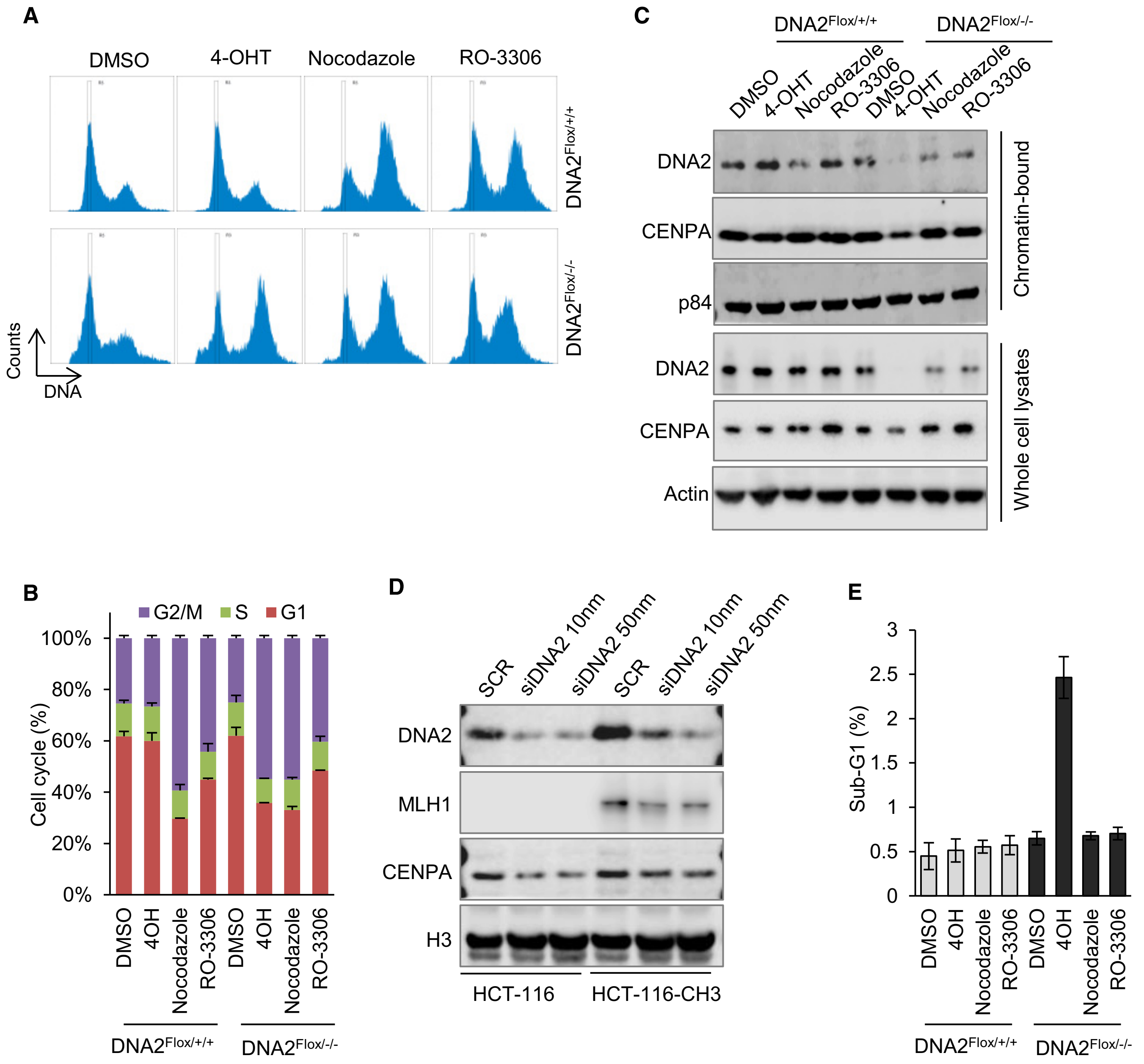

Figure EV4. The under-loading of CENP-A to chromatin in DNA2-null cells is not due to cell cycle changes and is not affected by MMR status.

A, B (A) DNA2$^{Flox/+/+}$ and DNA2$^{Flox/-/-}$ cells were incubated with 1 μM 4-OHT for 72 h, or 100 ng/ml nocodazole and 10 μM RO-3306 for 24 h which synchronize cells in G2 phase. Cells were harvested, fixed, stained with propidium iodide, and analyzed by flow cytometry to obtain cell cycle profiles. (B) The quantification of mean ± SD of the cell cycle distribution from three biological repeats.

C Western blot analysis of chromatin-bound proteins (after removal of cytoplasmic and nuclear chromatin-free fractions) extracted from cells treated as described in panel (A).

D The role of DNA2 in maintaining centromere integrity is not affected by MMR status. MMR-deficient HCT-116 and MMR-proficient HCT-116-CH3 cells were transfected with DNA2 siRNAs. Seventy-two hours after transfection, cells were harvested, and chromatin-bound proteins (top panel) or whole-cell lysates (bottom panel) were extracted for Western blot analysis of the indicated proteins. MLH1 (mutL homolog 1) was probed to confirm its expression status in the HCT-116 cells. CENP-A was checked as indicator of the centromere integrity.

E The mean ± SD of the cells in sub-G1 was from three replicates as in panel (A).

Source data are available online for this figure.

 © 2018 The Authors

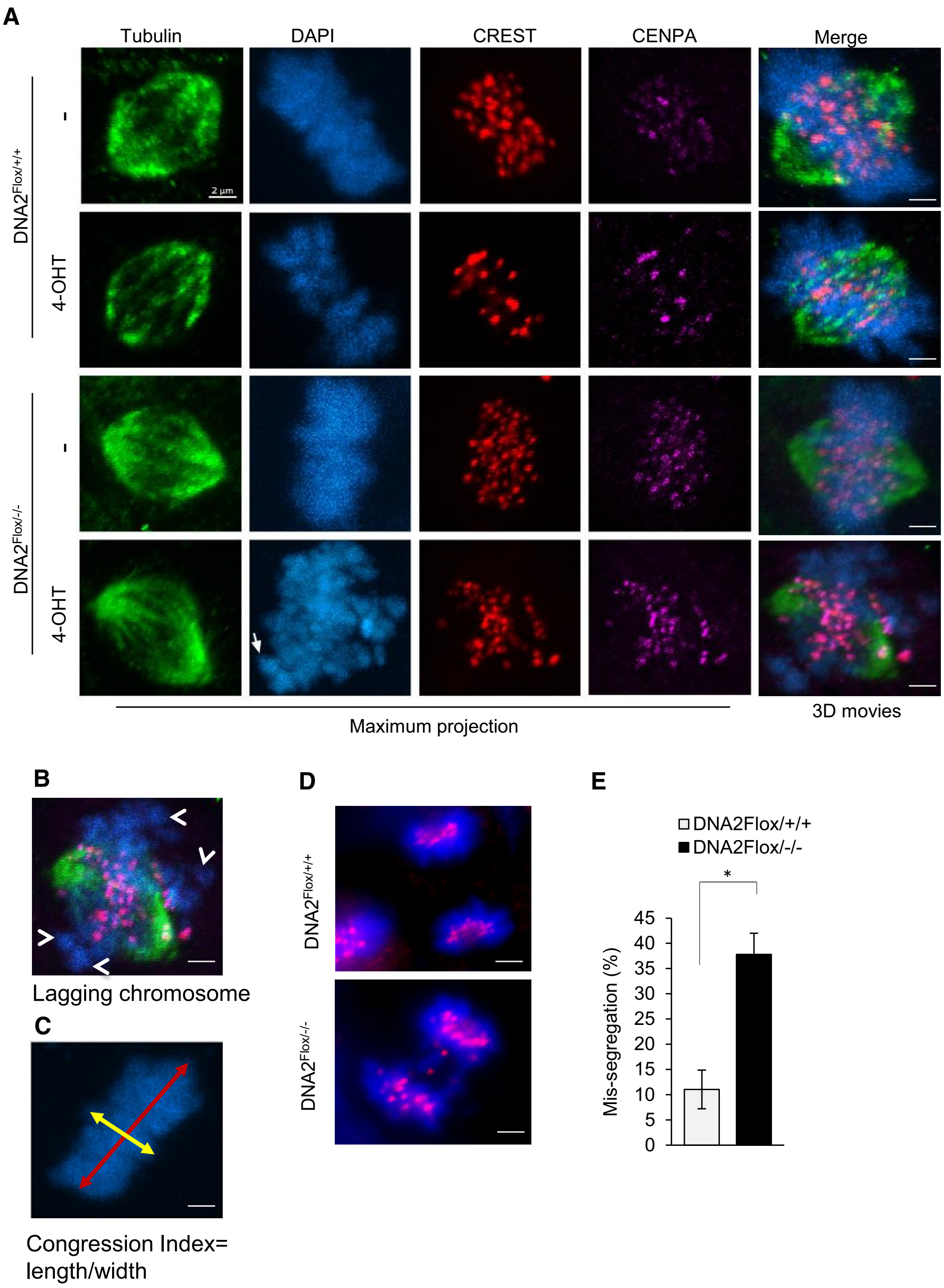

Figure EV5. Chromosome segregation defects in DNA2 null cells (see also Fig 4).

A Attachment of microtubules to the centromeres in WT and DNA2 null cells. Shown are the original images used to generate the merged images in Fig 4A. Movies of the 3D rotations of projections of these cells are shown on the right. Scale bars, 2 μm.

B, C Methodology for lagging chromosome (B) and congression index analysis (C). A lagging chromosome is defined as a cluster of DNA lacking anti-CREST and anti-CENP-A staining, as well as lack of attachment to α-tubulin (white arrows). The congression index was calculated as the ratio of metaphase chromosome width (parallel to the spindle poles) to length (perpendicular to the spindle poles). Scale bars, 2 μm.

D, E Microscopic analysis of chromosome segregation in the $DNA2^{Flox/+/+}$ and $DNA2^{Flox/-/-}$ cells. Panel (D) shows representative anaphase cells, which were analyzed by staining with DAPI (blue) and anti-CENP-A (red). Panel (E) shows the percentage of cells with segregation abnormalities (mean ± SD of three biological repeats, > 100 cells for each group). *P-value is < 0.05 using unpaired two-tailed t-test. Scale bars, 5 μm.

© 2018 The Authors

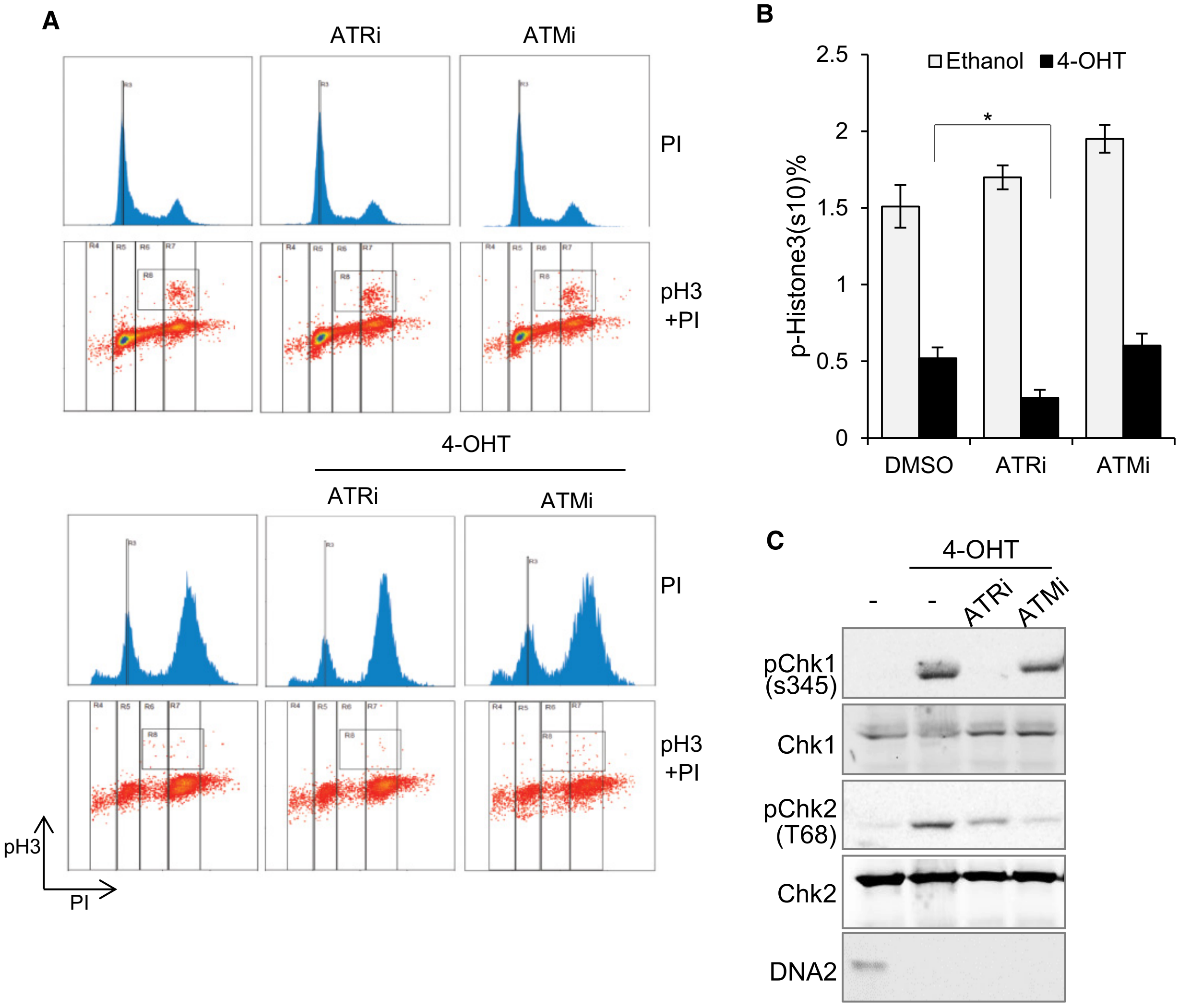

Figure EV6. Inhibition of ATR or ATM has distinct effects on the late-S arrest that is induced by DNA2 knockout.

A DNA2$^{Flox/-/-}$ cells were incubated with 4-OHT for 24 h and then cultured with specific inhibitors to ATR or ATM for another 24 h. Cells were collected, fixed, and stained with PI and phospho-histone H3 (S10) to assess cell cycle profile and mitotic entry.

B The mean ± SD of the percentages of phospho-histone H3-positive cells from three biological repeats (**P*-value is < 0.05 by unpaired two-tailed *t*-test).

C Western blotting confirming the inhibition of ATR or ATM. The efficiency of these specific inhibitors was confirmed by Western blotting: phospho-Chk1 on S345 for ATR kinase, and phospho-Chk2 at T60 for ATM kinase. Shown are representative images of three repeats.

© 2018 The Authors
