# Supplementary material for: hDNA2 nuclease/helicase promotes centromeric DNA replication and genome stability
Source: EMBO J. 2018 May 17;37(14):e96729. doi: 10.15252/embj.201796729 (PMC6043852; doi:10.15252/embj.201796729)
Supplement: Supplementary file 2 — Table EV1 [file EMBJ-37-e96729-s002.pdf]

Table EV1:
Primers used for QPCR in centromere and non-centromeric regions

Names of primers[1]	Primer sequences	Positions of primers[2]
Chr1 CEN-FW	CTCTGTTTATAAAGTCTGCAAGTGG	chr1:122525266
Chr1 CEN-Rev	CAACACAAGGGAAGTTTCTGAG	
Chr 1 ACTA1-FW	TGGTATCCTGAAACAAGCCAG	chr1:227563519
Chr 1 ACTA1-Rev	CCCATTCCACCTAACATCTTCG	
Chr 2 CEN-FW	GTGAGTATGACTTTGCAACACC	chr2:90379396
Chr 2 CEN-Rev	GGCTCATTGTTTCACCCATTG	
Chr 2 OCR4-FW	AGTCGGAGAGTCCAGTGG	chr2:147844517
Chr 2 OCR4-Rev	CTCACTCTTATCAGCTCGCC	
Chr3 CEN-FW	TCTGTCTAGTTTTATATGAAGAAATCACG	chr3:90673488
Chr3 CEN-Rev	TGTGCATTCAGCTTACGGAG	
Chr3 WDR6-FW	CTTCATAAACTTCAGCCCGC	chr3:49007204
Chr3 WDR6-Rev	CTTCCCCAAGAGGCCAC	
Chr 4 CEN-FW	ACTCTATAGTATCTGGAAGTGGACA	chr4:50485939
Chr 4 CEN-Rev	AGTTTCTGAGAATCCTTCTGTCTAG	
Chr4 GALNT7-FW	CTTGGGCCGGAAAACTAAC	chr4:173168753
Chr4 GALNT7-Rev	CCTGACTAAACCCTCCAAATCC	
Chr 5 CEN-FW	ACGATCCTTTACACAGAGCAG	chr5:47615381
Chr 5 CEN-ReV	TTTTCTACGAAGATATTTCCTTTTCTACT	
Chr 5 STC2-FW	CTTGGTTTCTTGGTGTCTGG	chr5:173314723
Chr 5 STC2-Rev	CTGGTATTGTAGGGAGTGGC	
Chr6 CEN-FW	AGACGGAAGCAATCTCAGAA	chr6:59072729
Chr6 CEN-Rev	TTCCAACGAAGGCCTCAAG	
Chr6 UBE3D-FW	GGGTGACAATAAATTGGGAACTG	chr6:82892468
Chr6 UBE3D-Rev	TCTGTTGCTCACGCCTAATAC	
Chr7 CEN-FW	CTGACAAGAGCAGAGAAGTGAG	chr7:62295163
Chr7 CEN-Rev	TGTCTCTCTCTGTCACCCATC	
Chr 7 TMEM209-FW	TTTCTTTGAATGTGGGCTTTGG	chr7:130164715
Chr 7 TMEM209-Rev	TCATCTACCTGATTCTTGTACCAAA	
Chr8 CEN-FW	GTTTGAAACCCTCTTTTTATAGTGTC	chr8:44333024
Chr8 CEN-Rev	ATGCTTCTGTCTAGTTTCTGTAGG	
Chr8 SORBS3-FW	ACTCACAAAACAGCCTGGAG	chr8:22551738
Chr8 SORBS3-Rev	CATGGAGAGTTGGTGCTGAG	
Chr 9 CEN-FW	TTCTTTGTGCTGTATGTCCTCA	chr9:44166056
Chr 9 CEN-Rev	TGAAGATATTCCCGTTTCCAAGG	
Chr9 SMC2-FW	CGGAGTCAAGCACAATTCG	chr9:104094260
Chr9 SMC2-Rev	TGCCTTCTACTTTTATCATACTCTACC	
Chr10 CEN-FW	ACACTCTTTGTAAGTCTGCAGG	chr10:40875763
Chr10 CEN-Rev	TCTAGCATTATATGAGGAAATCCCG	
Chr10 GHITM-FW	ATAGTTCAGTTGCAGAGCTCG	chr10:84139429
Chr10 GHITM-Rev	CTACCAGATTCCGTGTTCAGAC	
Chr11 CEN-FW	CAGCTCTGAAACCCTCTTATTCTAG	chr11:51506201
Chr11 CEN-Rev	ATGTGAAGATTTTCCTTTTCCACC	
Chr11 BUD13-FW	CTGTGGTCAAAACTGGCATTC	chr11:116748170
Chr11 BUD13-Rev	GAACTTGCCTACAAATGGAGTG	
Chr12 CEN-FW	TCAGCTCACCGAGTTTAACC	chr12:35072747
Chr12 CEN-Rev	CTCAAAGAGGTCCAAGTATCCAC	
Chr12 IFT81-FW	CGTCCTGGTTTTCATTGTTCC	chr12:110124335
Chr12 IFT81-Rev	GCAAAAGCAATTACTGTCCCTC	

[1]For each chromsome, two pairs of primers were designed. One was to amplify the centromeric DNA (names contain “CEN”) and another to an intron or exon of a gene.
[2]Location of primers was indicated according to hg38 from the UCSC genome browser database.

Table EV1 (continued): Primers used for QPCR in centromere and non-centromeric regions

Chr13 CEN-FW	TCCCTTTCACAGAGTAGGTTTG	chr13:16607611
Chr13 CEN-Rev	TTTCACCGTAGGCGTCAAG	
Chr13 NBEA-FW	GAGAATAAGCCTGCGGATCC	chr13:34942287
Chr13 NBEA-Rev	CAATGAGCCCCACTTCTCG	
Chr14 CEN-FW	CTCTTTCTGTAGTATCTGGATGTGG	chr14:17865008
Chr14 CEN-Rev	ACACATCACAAACTTGTTTCTCAG	
Chr14 DACT1-FW	CCTATTCGGAGACAGCTTTTCC	chr14:58634068
Chr14 DACT1-Rev	GCGGTGAACATTCTATTGCAG	
Chr15 CEN-FW	TCATAGAGTTGCACATTCCGG	chr15:19563367
Chr15 CEN-Rev	TTTCACCGTAGGCCTCAAAG	
Chr15 NTRK3-FW	CTGCCCTTCATACTCCACTTC	chr15:87876757
Chr15 NTRK3-Rev	AAGCCCATGTCCACTATCAAG	
Chr 16 CEN-FW	GATAGAGCCGTTTTGAAACACTG	chr16:34907651
Chr 16 CEN-Rev	TCCTTTTCAACCATAGGCCTC	
Chr16 CDH11-FW	CCCTGCCTTCATAACCGTAG	chr16:64946780
Chr16 CDH11-Rev	GTATCAGTACATGCCTAGACCTG	
Chr17 CEN-FW	AGCCTTCTCAGAAACTTCTCTG	chr17:26581913
Chr17 CEN-Rev	TCCCGTTTCCAAAGACATCTTC	
Chr17 ASIC2-FW	CCAAAGCAAACCTCAGAACATC	chr17:33013088
Chr17 ASIC2-Rev	CATGTCGTTCTTCTCTCCCTG	
Chr18 CEN-FW	GATACAGCACGTTGGAAACAC	chr18:15720094
Chr18 CEN-Rev	GGCTTGAATGCAGATATCACAAAG	
Chr18 SMAD2-FW	CAATGGAGAATCGCTTTTGGG	chr18:47833095
Chr18 SMAD2-Rev	GTGATATTAAGGAACGAGTGGGTAG	
Chr19 CEN-FW	CTGTTTGTAAAGTCTGCAAGTGG	chr19:25751640
Chr19 CEN-Rev	ACTGAGAATTCTTGTGTCTAGCAG	
Chr19 ZNF441-FW	CTGCGTCTCCCCAGATTG	chr19:11767000
Chr19 ZNF441-Rev	CACACAGACCACAACTCCTC	
Chr20 CEN-FW	CCTTCTCGGCAACTTGTTTG	chr20:26560385
Chr20 CEN-Rev	CCAACGAAATCCTCAAAGCTATG	
Chr20 TOP1-FW	ACGGTCGGGACTTAGTCTC	chr20:41028822
Chr20 TOP1-Rev	GTGATTTATACGGCTTTGCTCTG	
Chr21 CEN-FW	CGGAGTTGAACATTGCCTTTC	chr21:11149031
Chr21 CEN-Rev	CCTTTATCACCATGGGCCTC	
Chr21 SOD1-FW	TCCCTCTTTACTTCTCCCCAC	chr21:31659622
Chr21 SOD1-Rev	GCTCAGGGAAGGTGGAAAAC	
Chr22 CEN-FW	CCCTCATATCCCAAACATCACC	chr22:15764366
Chr22 CEN-Rev	TGGAGAAAATAGAGCAAGTGGAG	
Chr22 CABIN1-FW	GTTCCCAGTAACCGCAGG	chr22:24011315
Chr22 CABIN1-Rev	CGGTCCCACACTTATAACCTG	
ChrX CEN-FW	GTGACGATGGAGTTTAACTCAGGG	chrX: 62,474,280
ChrX CEN-Rev	TGCTTCCGTTCAGTTATGGGAAG	
ChrX SERPINA7-FW	TGACTCTGGAGTGATTCTAGGT	chrX:106033199
ChrX SERPINA7-Rev	TTCTGCCACATATGACCTTGG	
ChrY CEN-FW	GTATTCCACTTCATTCCCCTCC	chrY:10659172
ChrY CEN-Rev	TGATTGGAGTGAAAATTGGGAAAG	
ChrY FAM41AY1-FW	TCTTACTGGCAAGAACACCTG	chrY:17500958
ChrY FAM41AY1-FRew	GCTCACTCCCCTTTCTACAC	
hBG-FW	GGTGAAGGCTCATGGCAAGA	chr11:5247864
hBG-Rev	AAAGGTGCCCTTGAGGTTGTC	

[1]For each chromsome, two pairs of primers were designed. One was to amplify the centromeric DNA (names contain “CEN”) and another to an intron or exon of a gene.
[2]Location of primers was according to hg38 from the UCSC genome browser database.
