# Supplementary figures and images for: hDNA2 nuclease/helicase promotes centromeric DNA replication and genome stability
Source: EMBO J. 2018 May 17;37(14):e96729. doi: 10.15252/embj.201796729 (PMC6043852; doi:10.15252/embj.201796729)

**Fig EV1A**

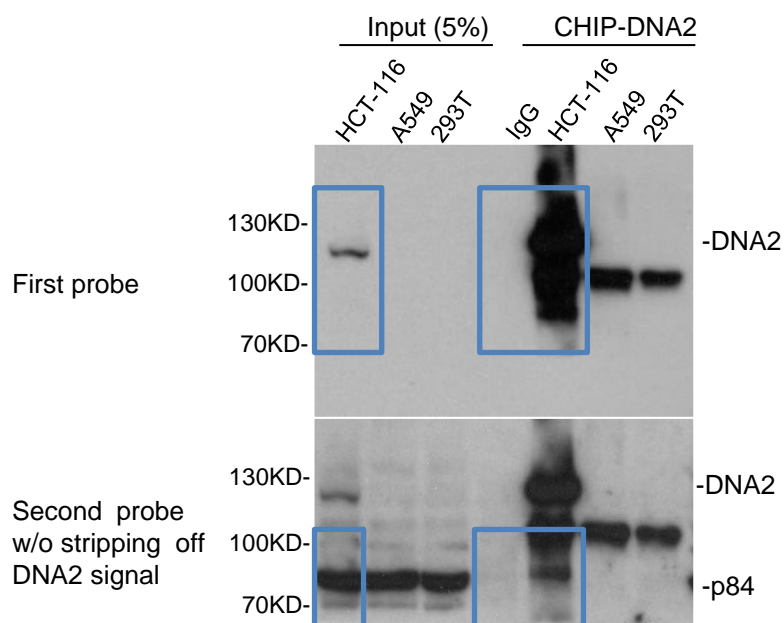

**Fig EV1B**

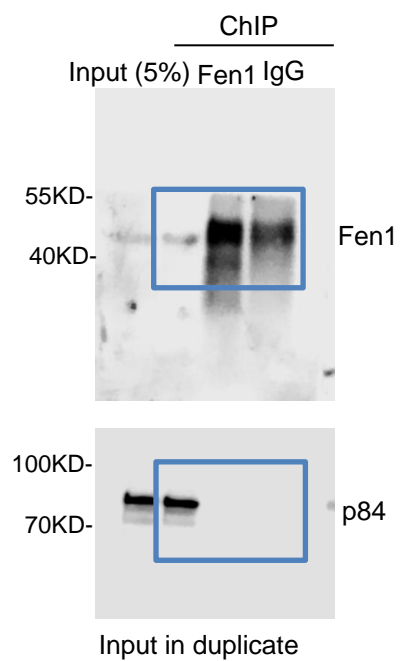

**Fig EV1C**

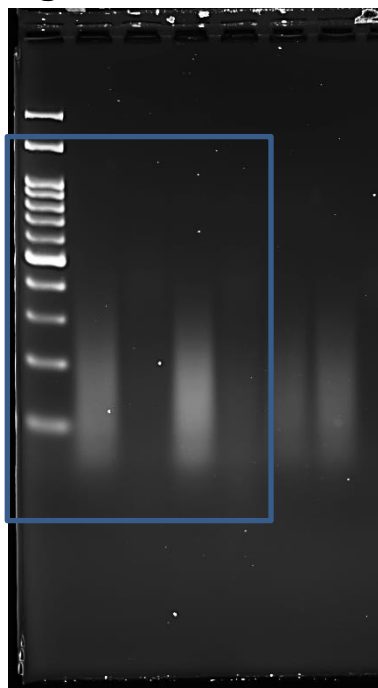

Supplement: Supplementary file 3 — Source Data for Expanded View [file EMBJ-37-e96729-s006.zip › EMBOJ_96729_Source_data_fig_EV1.pdf]

**Fig EV3B**

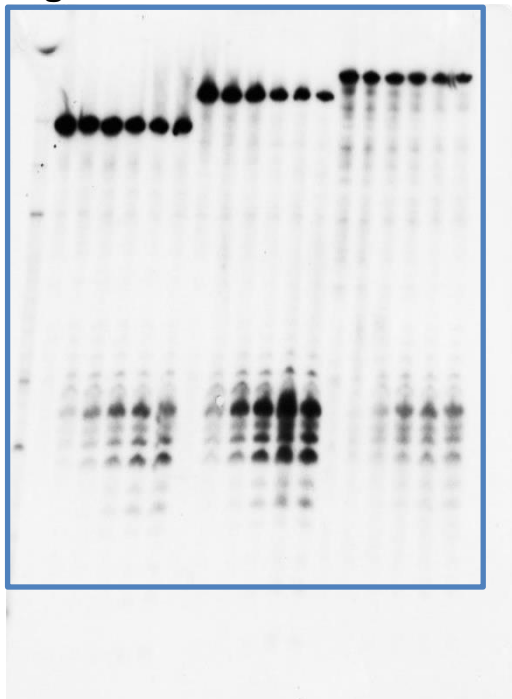

**Fig EV3C**

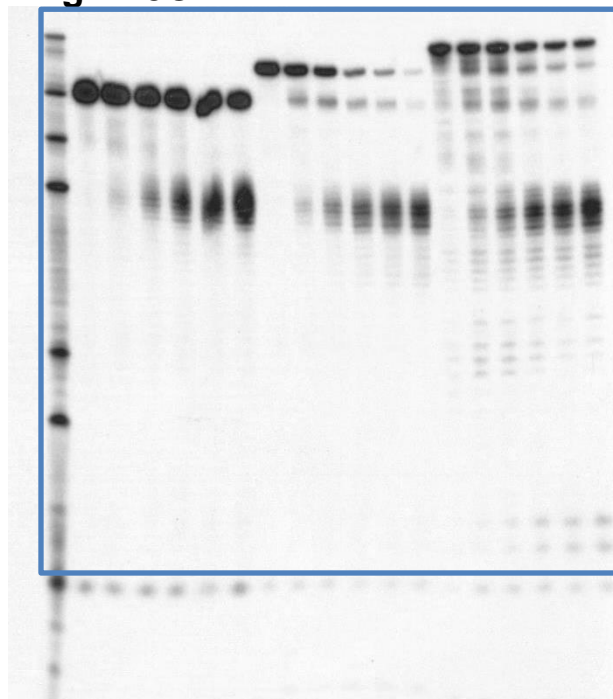

**Fig EV3E**

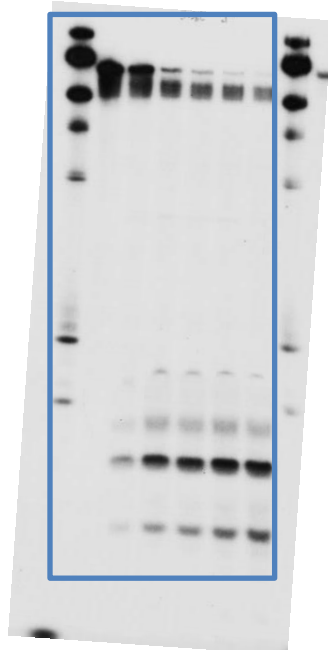

**Fig EV3F**

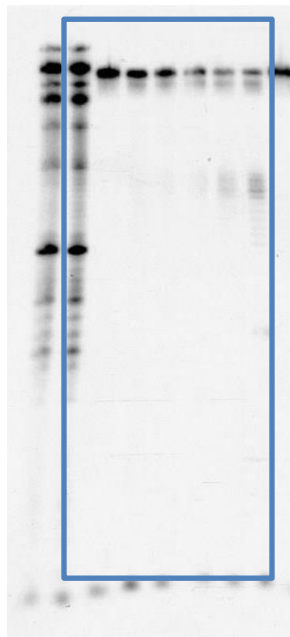

Supplement: Supplementary file 3 — Source Data for Expanded View [file EMBJ-37-e96729-s006.zip › EMBOJ_96729_Source_data_fig_EV3.pdf]

Fig EV4C

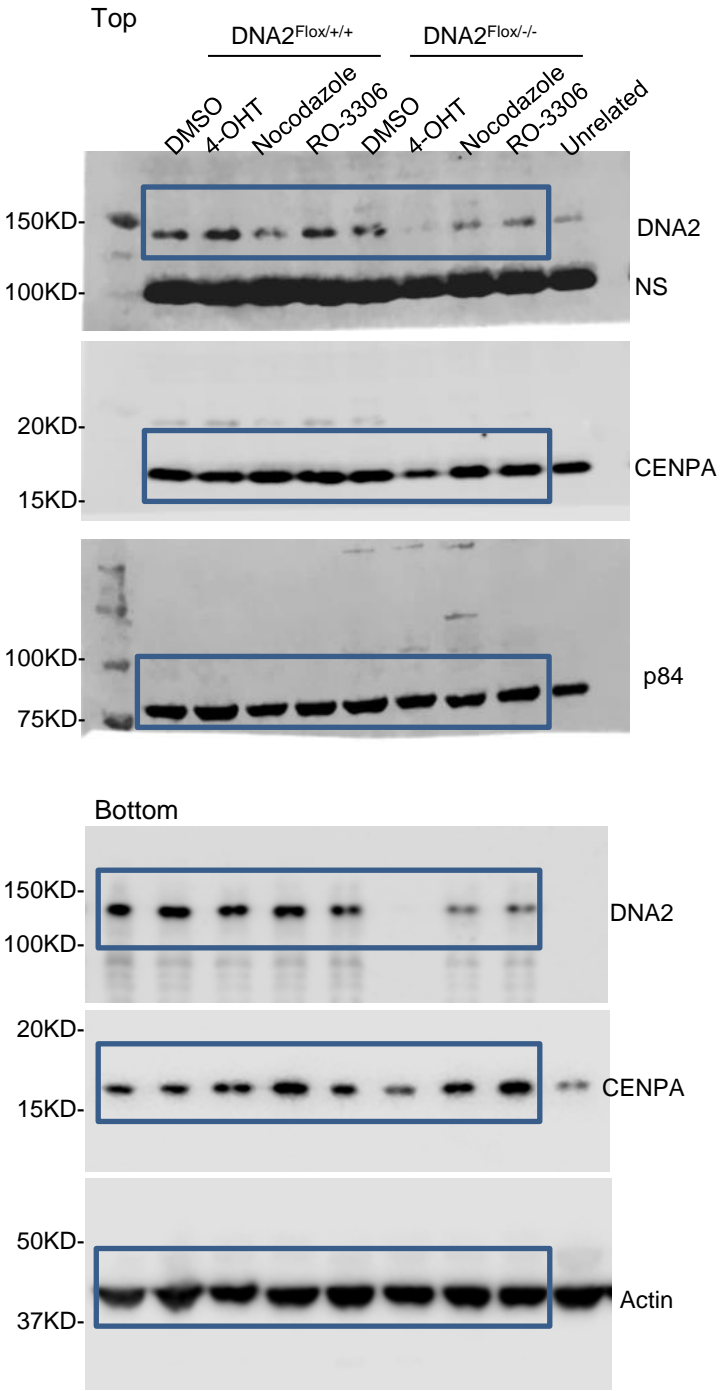

Fig EV4D

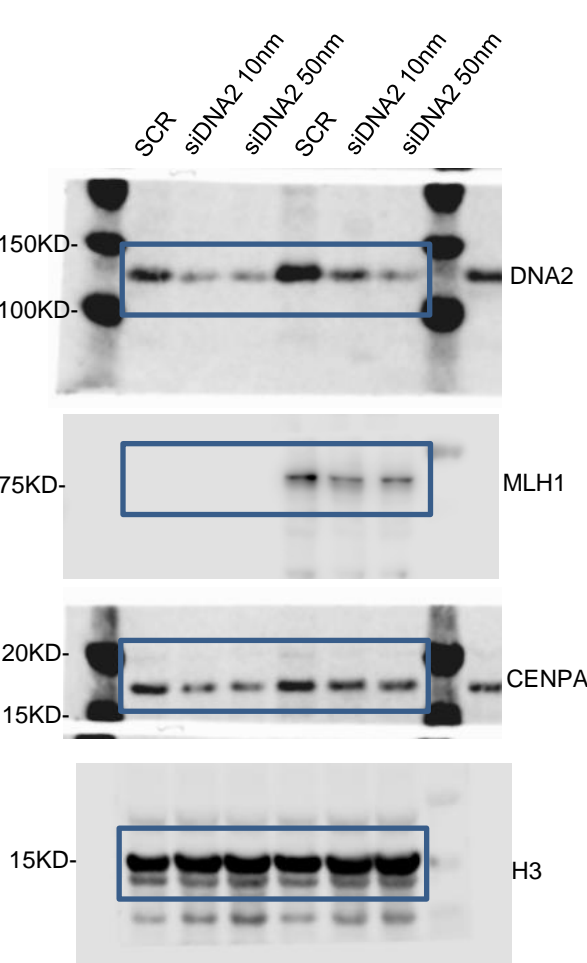

Supplement: Supplementary file 3 — Source Data for Expanded View [file EMBJ-37-e96729-s006.zip › EMBOJ_96729_Source_data_fig_EV4.pdf]

**Fig 2D**    5' labeled substrates

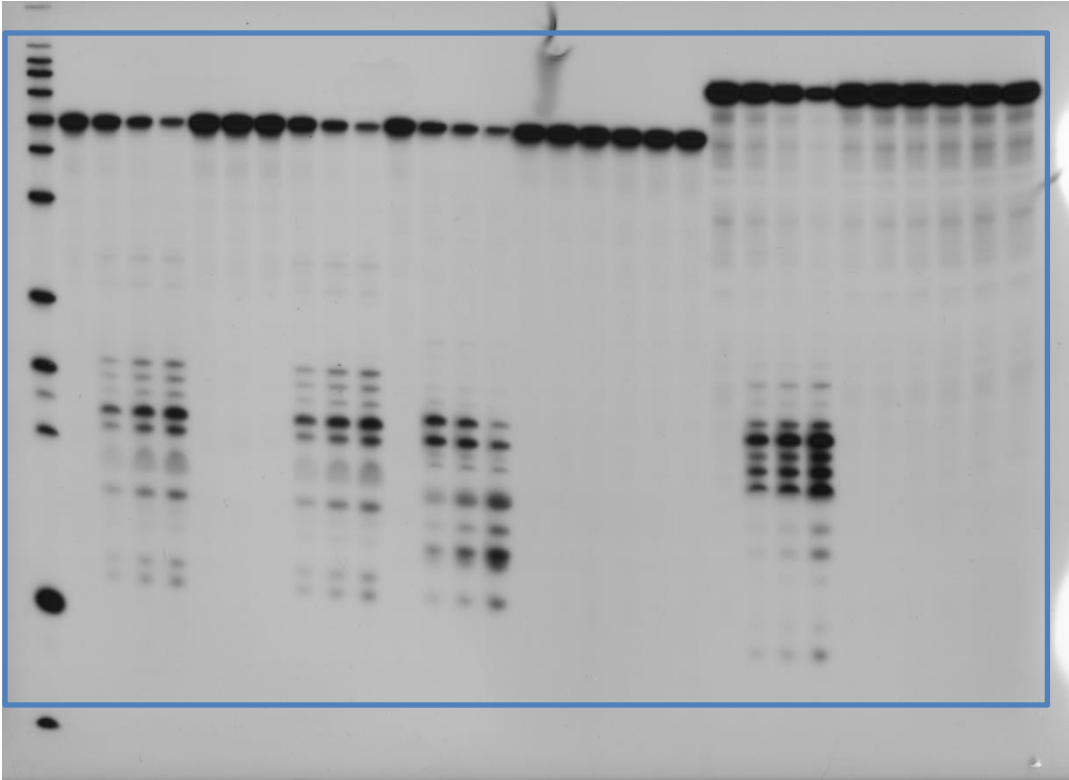

**Fig 2E**    3' labeled substrates

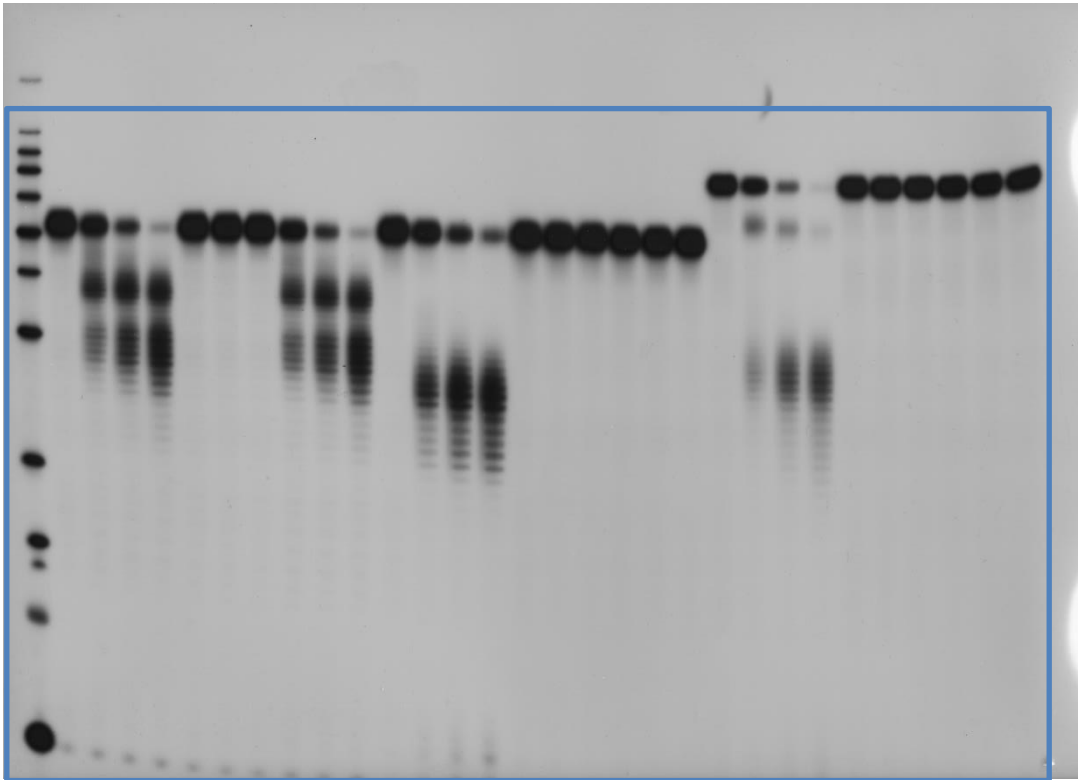

Supplement: Supplementary file 5 — Source Data for Figure 2 [file EMBJ-37-e96729-s003.pdf]

**Fig 4 B**

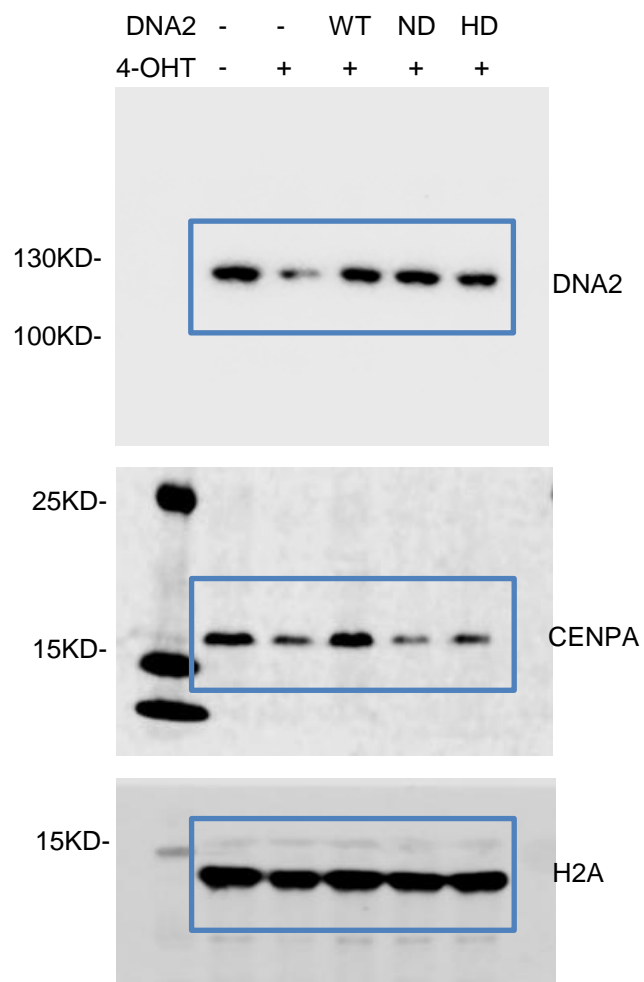

Supplement: Supplementary file 6 — Source Data for Figure 4 [file EMBJ-37-e96729-s004.pdf]
