# Supplementary material for: hDNA2 nuclease/helicase promotes centromeric DNA replication and genome stability
Source: EMBO J. 2018 May 17;37(14):e96729. doi: 10.15252/embj.201796729 (PMC6043852; doi:10.15252/embj.201796729)
Supplement: Supplementary file 7 — Source Data for Figure 7 [file EMBJ-37-e96729-s005.pdf]

Fig 7A

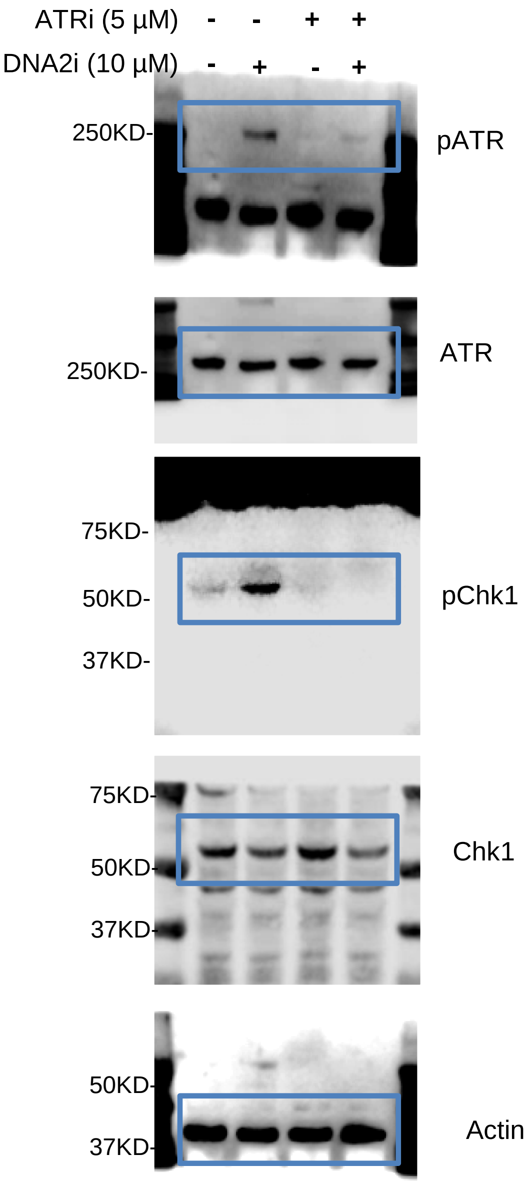
ATRi (5 µM) - - + +
DNA2i (10 µM) - + - +
250KD-
pATR
250KD-
ATR
75KD-
50KD-
37KD-
pChk1
75KD-
50KD-
37KD-
Chk1
50KD-
37KD-
Actin
